# The short-term association between environmental variables and daily pediatric asthma patient visits in Hangzhou, China: A time-series study

**DOI:** 10.1016/j.heliyon.2024.e37837

**Published:** 2024-09-13

**Authors:** Yuqing Feng, Xin Yang, Yingshuo Wang, Lei Wu, Qiang Shu, Haomin Li

**Affiliations:** a Department of Data and Information, Children's Hospital, Zhejiang University School of Medicine, National Clinical Research Center for Child Health, National Children's Regional Medical Center, Hangzhou, 310052, China; b Department of Pulmonology, Children's Hospital, Zhejiang University School of Medicine, National Clinical Research Center for Child Health, National Children's Regional Medical Center, Hangzhou, 310052, China; c Department of Endoscopy Center, Children's Hospital, Zhejiang University School of Medicine, National Clinical Research Center for Child Health, National Children's Regional Medical Center, Hangzhou, 310052, China; d Department of Genetics and Metabolism, Children's Hospital, Zhejiang University School of Medicine, National Clinical Research Center for Child Health, National Children's Regional Medical Center, Hangzhou, 310052, China

**Keywords:** Meteorological variables, Air pollutants, Child, Asthma, Hospital visits. relative risk

## Abstract

**Background:**

To date, a large number of studies have shown correlations between environmental variables and pediatric asthma in short-term lag time. However, their results are inconsistent. Therefore, we aimed to evaluate the short-term impact of environmental variables on daily pediatric asthma patients’ visits (DPAPV) in Hangzhou, China, and find the most important risk factor.

**Methods:**

Generalized additive distribution lag non-linear model (GAM-DLNM) was applied to explore the effect of environmental variables on DPAPV in single- and multi-variable models in Hangzhou, China from 2014 to 2021. Then, risk factors of pediatric asthma were selected (p < 0.05 both in single- and multi-variable models) and used weighted quantile sum (WQS) regression model to evaluate their relative importance.

**Results:**

There were 313,296 pediatric asthma patient records between 2014 and 2021. Both in single- and multi-variable models, PM_2.5_, PM_10_, and NO_2_ exhibited significant positive correlations in short-term lag time and these correlations reached their maximum in lag day 2 (RR = 1.00, 95%CI:1.00 to 1.01), lag day 2 (RR = 1.00, 95%CI:1.00 to 1.01), and lag day 3 (RR = 1.04, 95%CI:1.02 to1.05), respectively. The WQS index showed that NO_2_ had the greatest relative importance (weight over 70 %). The relative importance of NO_2_ increased with time passing. Males were more susceptible to the adverse effects of NO_2_.

**Conclusion:**

PM_2.5_, PM_10_, and NO_2_ had significant adverse effects on pediatric asthma. Among them, NO_2_ presented the greatest and most important adverse effect on the disease. Therefore, parents could give priority to paying attention to NO_2_ to control children's asthma.

## 1 Introduction

It is well known that asthma is one of the most common respiratory diseases. Compared with adults, children are more susceptible to asthma due to their lungs are not fully developed and their immunities are weakened [1]. Therefore, we should be vigilant for asthma in children. China is one of the worst countries for pediatric asthma. From 2009 to 2010, the Chinese government carried out a survey about the number of children with asthma in 43 cities. The result showed that there were about 10 million children with asthma among urban children in China, and the prevalence rate varied between cities (2.0–4.2 %) [2]. Pathophysiologically, long-term airway inflammation is one of the causes of asthma symptoms. Therefore, control of inflammation becomes the focus of asthma control [3]. Unfortunately, the control of inflammation in pediatric asthma remains a challenge in China [4]. Thus, it is necessary to explore the predisposing factors of pediatric asthma to effectively control it.

Exposure environment is an important factor in pediatric asthma. Over the past two decades, pollutants in China have undergone a process of growth and then gradual improvement, with industrial waste gas pollution emissions and urban air pollution index slowly decreasing in numerous Chinese cities from 2015 [5,6]. The long-term effect has also been accompanied by a subsequent fluctuation in the number of children with asthma. However, the short-term associations among them were still inconsistently reported in China. A study in Shanghai showed that PM, SO_2,_ and NO_2_ were all significantly positively associated with the risk of pediatric asthma [7]. However, the previous study in Xi'an found that the association between NO_2_ and the risk of this disease was not significant [8]. Meanwhile, another study in 43 cities also found that NO_2_ and PM had no significant association with it [9]. These inconsistent results may be caused by variations in air pollution levels, economic levels, medical levels, and population characteristics in different regions. In addition, many studies also ignored lag effects and complex high autocorrelations of these exposure variables in real environments. Therefore, these results may not be representative.

Hangzhou is located in the subtropical monsoon climate zone with various climatic conditions. It is the capital city of Zhejiang Province with about 12 million residents in 2021. In this study, we applied a generalized additive distribution lag non-linear model (GAM-DLNM) and weighted quantile sum (WQS) regression model to evaluate the effects of environmental variables on the daily number of pediatric asthma patients’ visits (DPAPV) in Hangzhou to offer reasonable recommendations for parents and patients to effectively control pediatric asthma according to the result of our study. The GAM-DLNM was applied to explore the effect of these environmental variables with the lag effect, and the WQS regression model was used to screen for high-risk factors in high auto-associated variables.

## 2 Material and method

### 2.1 Data collection

#### 2.1.1 Patient data

DPAPV data was from Children's Hospital, Zhejiang University School of Medicine, Hangzhou, Zhejiang, between January 1, 2014, and December 31, 2021. This hospital is the center of children's health care in Zhejiang Province and a National Clinical Research Center for children's health and diseases in China. Its pediatrics department has more than 3 million outpatient visits and more than 70 thousand inpatient admissions each year.

**Fig. 1:**
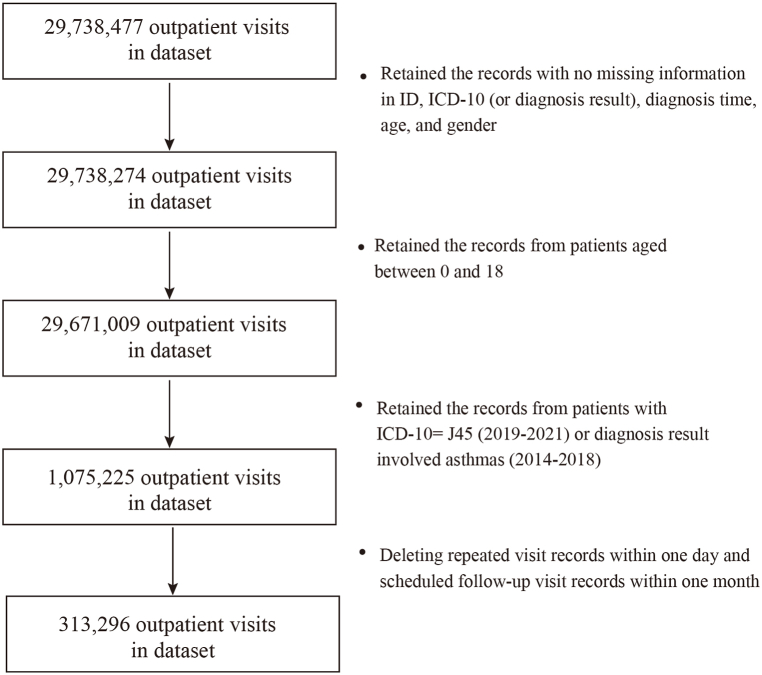
The flow diagram of the data screening.

Obtained DPAPV data containing ID, name, diagnosis time, diagnosis result, diagnosis code, age, gender, and birthday. The code principles for diagnosis code in the data are International Classification of Diseases, Revision 10 (ICD-10). Before 2018, this hospital did not require doctors to use ICD-10 in EHR, so we screened patient records from 2014 to 2018 based on whether the diagnosis result involved asthma. In this study, patients with ICD-10 code J45 (or diagnosis result involved asthma) and ages between 0 and 18 years were regarded as pediatric asthma patients. There are 313,296 valid visit records (without lost ID, diagnosis result or ICD-10, diagnosis time, age, and gender) after deleting repeated visit records within one day and scheduled follow-up visit records within one month. Fig. 1 was the flow diagram of the data screening.

This study was approved by the Institutional Review Board of the Children's Hospital of Zhejiang University School of Medicine (2018-IRB-046), and the requirement for informed consent was waived.

#### 2.1.2 Meteorological data and pollution data

Daily 24-h mean of temperature, humidity, precipitation, wind speed, visibility, and sea level pressure were downloaded from the National Center for Environmental Information (https://www.ncei.noaa.gov). As an agency of the United States government, it manages one of the largest extensive archives of environmental data in the world.

Daily 24-h mean concentrations of particulate matter of 2.5 μm or less in diameter (PM_2.5_), particulate matter of 10 μm or less in diameter (PM_10_), nitrogen dioxide (NO_2_), sulfur dioxide (SO_2_), carbon monoxide (CO), and air quality index (AQI) were taken from the national air pollution prevention and control monitoring network. It is network has 1436 monitoring stations (including 135 control monitoring stations) in 338 cities in China and there are 10 stations in Hangzhou.

Both the meteorological data and pollutant data for the aforementioned environmental variables had no missing data points. From January 1, 2014, to December 31, 2021, a total of 2922 records were available for each of the environmental variables because of 2922 monitor days.

### 2.2 Statistical analysis

Descriptive statistics were used to summarize the distribution of DPAPV and environmental variables during the study period. Then, Spearman's rank correlation coefficients were used to evaluate the correlations between meteorological variables and air pollutants.

GAM-DLNM analysis was used to evaluate the short-term lag effect on the association between environmental variables and DPAPV. It was proposed by Gasparrini et al., in 2017 [10]. The basic model of the GAM-DLNM is as follows:

Yt∼Quasi−Poisson(μt)

log(μt)=α+cb∑(Meteorologicalvariables/Airpollutans,lag)+factor(stratum)

where Yt denotes the DPAPV on the day t. α was the intercept. cb(Weather/Airpollution,lag) represented the cross-basis function of each environmental variable. factor(stratum) was a time stratum variable and contained year, month, and weekday, and it was used to control the potential temporal confounding effects. The result of this model was the RR of the DPAPV, and it denoted the effect of these meteorological variables or air pollutants on DPAPV when air pollutants increased by 10 units (CO increased by 0.1 mg/m^3^), respectively. In addition, medium values of environmental variables were set as the control groups. In single-variable models, significant variables (p < 0.05) were selected and regarded as related variables.

Considering the potential interactions between related variables, GAM-DLNM was also created to cover all selected related variables to find important variables significantly associated with DPAPV in multi-variable models. According to the report of PM_2.5_ composition in Hangzhou, SO_2_ and NO_x_ were important raw materials for the generation of PM_2.5_^9^. In addition, the composition of PM_10_ contains PM_2.5_. Considering these relationships, NO_2_ and SO_2_ were removed from multi-variable models for PM_10_. Similarly, in the multi-variable model for PM_2.5_, PM_10_, NO_2_ and SO_2_ were also removed.

Weighted quantile sum (WQS) regression model was used to evaluate the relative importance of selected important variables from GAM-DLNM in the short-term lag time [11]. It is the model that evaluates the risk factors among highly correlated chemicals. It models the mixture effect of the entire index by using a weighted index of numerous correlated chemical exposures, and assigns weights to each component in the mixture to represent its relative importance [12]. In detail, based on the quantile of chemical composition, it divides these features into two groups according to the positive and negative effects. It assumes that all the positive features of the index act in the same direction, while all the negative features of the index act in the same and opposite direction to the positive features. It created a weighted index to represent the relevant chemical mixtures (the sum of weight is equal to 1, and the individual weight is between 0 and 1). The greater index the more important chemicals. In this study, there were 60 % training data and 40 % validation data in the WQS model based on the previous studies [13,14]. 100 bootstrap samples were set to increase the sensitivity of the model. It can also be applied in environmental epidemiology to evaluate the overall mixture effect of environmental variables and identify the most important variable. In this study, we constructed the WQS model by percentage of selected variables and estimated the importance of the important risk factors in different sub-groups. All analyses were performed in R version 4.0.2.

## 3 Result

### 3.1 Data description

As shown in Table 1, a total of 313,296 visits for pediatric asthma were recorded between January 1, 2014, and December 31, 2021. Of these records, 204,313 (65.21 %) were boys, and 108,983 (34.79 %) were girls. There were three age groups, Group 1 represented the children aged below 1, Group 2 represented the children aged between 1 and 12, and Group 3 represented the children aged between 13 and 17. Among the three age groups, it was obvious that the children in Group 2 were most affected (287,434 records, 91.75 %). The annual number of asthma visits showed a yearly declining trend, except for 2015 to 2016 (increased by 1,832, 0.59 %), and 2020 to 2021 (increased by 5,404, 1.73 %). The former might be influenced by the opening of the new sub-hospital at the end of 2014 which could increase the maximum daily outpatient visits and attract more citizens near the new sub-hospital. The latter might be influenced by the end of the COVID-19 pandemic at the end of 2019 because China adopted a policy of home isolation during the pandemic between 2019 and 2020.

**Table 1:** Descriptive statistics of the number of daily visits with pediatric asthma between January 1, 2014, and December 31, 2021.

Characteristics	Number of patients	Percentage (%)
**Sex**		
**Boys**	204,313	65.21
**Girls**	108,983	34.79
**Age (in year)**		
**Group1(< 1)**	15,633	4.99
**Group2(1–12)**	287,434	91.75
**Group3(13–17)**	10,229	3.26
**Year**		
**2014**	47,012	15.01
**2015**	46,350	14.79
**2016**	48,182	15.38
**2017**	43,428	13.86
**2018**	40,457	12.91
**2019**	37,151	11.86
**2020**	22,656	7.23
**2021**	28,060	8.96
**Total**	313,296	100

Table S1 in the supplemental showed descriptive statistics of daily meteorological variables, and air pollutant concentrations between January 1, 2014, and December 31, 2021. Their mean values were lower than the levels of ambient air quality standards in China (GB 3095-2012). Fig. S1 in the supplemental presented the daily distribution of meteorological variables, and air pollutant concentrations. Distributions of 6 meteorological variables and 6 air pollutants showed similar periodicity. The difference was that the value of meteorological variables kept relative stability, but the level of air pollutants decreased yearly (except NO_2_). This means atmospheric quality has improved in Hangzhou these years.

### 3.2 Spearman's rank correlation coefficients

**Fig. 2:**
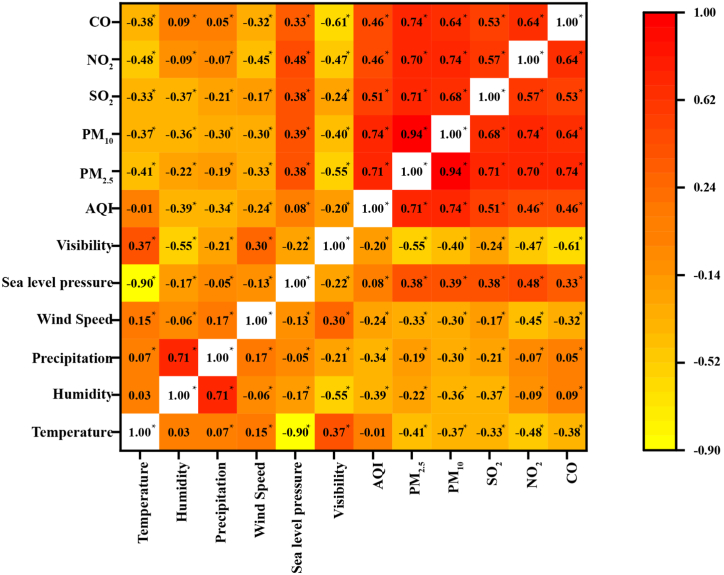
Spearman's rank correlation coefficients of meteorological variables and air pollutants.

Fig. 2 showed Spearman's rank correlation coefficients (r) between air pollutants and meteorological variables. There were highly positive correlations (|r|>0.4,p<0.05) among air pollutants. Compared with other air pollutants, PM_10_ and PM _2.5_ had the highest correlation (r=0.94,p<0.05) due to PM_10_ containing PM_2.5_. NO_2_ and SO_2_ also had highly positive correlations with PM (PM_2.5_ and PM_10_) (|r|>0.6,p<0.05). In addition, compared with coefficients among air pollutants, coefficients between meteorological variables and air pollutants were relatively low. Correlations between environmental variables (meteorological variables and air pollutants) and DPAPV were showed in Table S2.

### 3.3 Distributed lag nonlinear model analyses

#### 3.3.1 Single-variable model

**Fig. 3:**
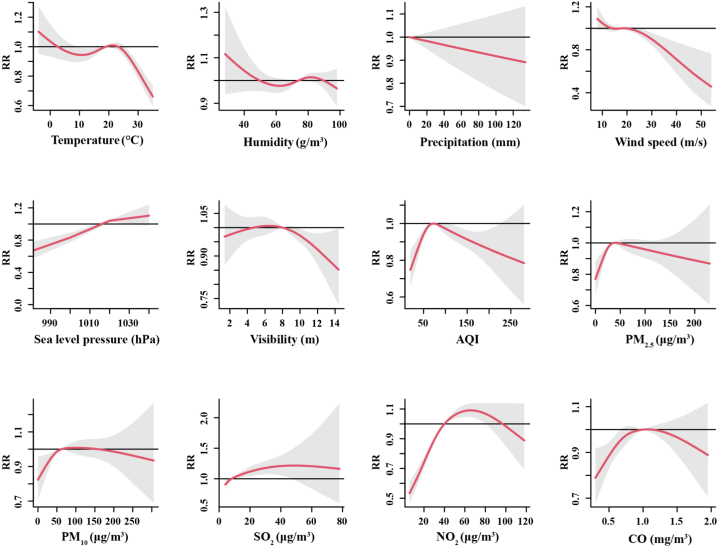
Overall cumulative association lagged 0–6 days of each environmental variable in single-variable GAM-DLNM.

Table 2 listed the lag-specific associations of meteorological variables and air pollutants with DPAPV in single-variable models. Each 10-unit increase in temperature and wind speed reduced the relative risk (RR) of the DPAPV (RR < 1, p < 0.05) on different lag days, respectively. Temperature only showed a significant negative effect on lag day 5 (RR = 0.96, 95%CI: 0.95 to 0.98, p < 0.05), and wind speed only on lag day 1 (RR = 0.98, 95%CI: 0.97 to 0.99, p < 0.05). On the contrary, each 10-unit increase in humidity, sea level pressure, PM_2.5_, PM_10_, SO_2_, and NO_2_ all increased the RR of DPAPV (RR > 1, p < 0.05) on different lag days. Among them, sea level pressure and NO_2_ kept relatively long-term significant positive effects on DPAPV. Sea level pressure lasted for 5 days (from lag day 0 to lag day 4), and the largest RR appeared on lag day 0 (RR = 1.02, 95%CI: 1.00 to 1.04, p < 0.05). NO_2_ lasted for 4 days (from lag day 2 to lag day 5), and the largest RR appeared on lag day 3 (RR = 1.02, 95%CI: 1.01 to 1.02, p < 0.05). Fig. 3 showed the overall cumulative association of each environmental variable in single-variable models lagged 0–6 days.

**Table 2:** Lag effect of each environmental variable on DPAPV in single-variable DLNM.

Variable	Median	RR (95%CI)						
		Lag day 0	Lag day 1	Lag day 2	Lag day 3	Lag day 4	Lag day 5	Lag day 6
**Temperature**	19.10	0.98(0.95,1.02)	0.99(0.98,1.01)	0.99(0.98,1.01)	0.99(0.97,1.02)	0.98(0.96,1.00)	**0.96(0.95,0.98)∗**	0.95(0.91,0.98)
**Humidity**	74.02	1.00(0.99,1.01)	1.00(0.99,1.00)	1.00(1.00,1.01)	1.00(1.00,1.01)	1.00(1.00,1.01)	**1.01(1.00,1.01)∗**	1.01(1.00,1.02)
**Precipitation**	0.00	0.99(0.99,1.00)	1.00(0.99,1.00)	1.00(0.99,1.00)	1.00(1.00,1.00)	1.00(1.00,1.00)	1.00(1.00,1.01)	1.00(1.00,1.01)
**Wind speed**	18.00	0.97(0.96,0.99)	**0.98(0.97,0.99)∗**	0.99(0.98,1.00)	0.99(0.98,1.00)	1.00(0.99,1.01)	1.00(0.99,1.01)	1.01(0.98,1.02)
**Sea level pressure**	1016.50	**1.02(1.00,1.04)∗**	**1.02(1.01,1.03)∗**	**1.01(1.01,1.02)∗**	**1.01(1.00,1.02)∗**	**1.01(1.00,1.02)∗**	1.01(1.00,1.01)	1.00(0.99,1.02)
**Visibility**	7.70	1.08(0.95,1.23)	1.03(0.96,1.11)	0.99(0.92,1.06)	0.95(0.87,1.04)	0.92(0.86,0.99)	0.90(0.83,0.96)	0.87(0.77,0.99)
**AQI**	74.00	1.00(1.00,1.01)	1.00(1.00,1.00)	1.00(1.00,1.00)	1.00(1.00,1.00)	1.00(1.00,1.00)	1.00(0.99,1.00)	1.00(0.99,1.00)
**PM**_**2.5**_	36.00	1.01(1.00,1.01)	**1.00(1.00,1.01)∗**	1.00(1.00,1.00)	1.00(0.99,1.00)	1.00(0.99,1.00)	1.00(0.99,1.00)	0.99(0.99,1.00)
**PM**_**10**_	62.00	1.00(1.00,1.01)	**1.00(1.00,1.01)∗**	1.00(1.00,1.00)	1.00(1.00,1.00)	1.00(1.00,1.00)	1.00(1.00,1.00)	1.00(0.99,1.00)
**SO**_**2**_	8.00	**1.04(1.01,1.07)∗**	**1.03(1.02,1.05)∗**	**1.03(1.01,1.04)∗**	1.02(1.00,1.03)	1.00(0.99,1.02)	0.99(0.98,1.01)	0.98(0.97,1.01)
**NO**_**2**_	19.10	1.00(0.99,1.01)	1.01(1.00,1.01)	**1.01(1.01,1.02)∗**	**1.02(1.01,1.02)∗**	**1.01(1.01,1.02)∗**	**1.01(1.00,1.02)∗**	1.00(0.99,1.02)
**CO**	74.02	1.00(0.99,1.00)	1.00(0.99,1.00)	1.00(0.99,1.00)	1.00(0.99,1.00)	1.00(0.99,1.00)	1.00(0.99,1.00)	1.00(0.99,1.01)

Note: **∗** means p < 0.05; These results are presented the RR of DPAPV in single-variable models when 10.0 μg/m^3^ increase for PM_2.5_, PM_10_, SO_2_, NO_2_, 0.1 mg/m^3^ increase for CO, 10 °C increase for temperature, 10 g/m^3^ increase for humidity, 10 mm increase for precipitation, 10 m/s increase for wind speed, 10 hPa increase for sea level pressure, 10m increase for visibility, and 10 increase for AQI.

#### 3.3.2 Multi-variable model

According to Table 2, temperature, humidity, wind speed, sea level pressure, PM_2.5_, PM_10_, SO_2,_ and NO_2_ had significant associations with the RR of the DPAPV, and they were used to create multi-variable models.

As shown in Table 3, in multi-variable models, only PM_2.5_, PM_10,_ and NO_2_ still presented significant positive associations with the RR of DPAPV (RR > 1, p < 0.05). Therefore, PM_2.5_, PM_10_, and NO_2_ were important pollutants that influent the DPAPV. Interestingly, among them, the significant effect of NO_2_ still lasted for a relatively longer time (lag day 1 to lag day 5). Its greatest RR appeared in lag day 3 (RR = 1.04, 95%CI:1.02 to 1.05, p < 0.05). Compared with single-variable models (in Table 2), the effect of NO_2_ on RR for DPAPV had elevated in multi-variable models. However, the effects of PM_2.5_ and PM_10_ were still slight on RR for DPAPV kept relatively stable in the multi-variable model. Overall, PM_2.5_, PM_10_, and NO_2_ were important factors affecting the RR of DPAPV, and NO_2_ had a greater and more long-lasting effect than PM.

**Table 3:** Lag effect of each related environmental variable on DPAPV in multi-variable DLNM.

Variable	Median	RR (95%CI)						
		Lag day 0	Lag day 1	Lag day 2	Lag day 3	Lag day 4	Lag day 5	Lag day 6
**Temperature**	19.10	1.00(0.96,1.05)	1.00(0.98,1.02)	1.00(0.98,1.03)	1.00(0.97,1.04)	1.00(0.98,1.03)	1.00(0.97,1.02)	0.99(0.94,1.04)
**Humidity**	74.02	0.98(0.97,1.00)	0.99(0.98,1.00)	1.00(0.99,1.00)	1.00(0.99,1.01)	1.00(0.99,1.01)	1.00(0.99,1.00)	0.99(0.98,1.01)
**Wind speed**	18.00	1.00(0.98,1.02)	1.00(0.99,1.02)	1.01(1.00,1.02)	1.01(1.00,1.03)	1.01(1.00,1.03)	1.01(1.00,1.02)	1.01(0.99,1.03)
**Sea level pressure**	1016.50	1.00(0.97,1.02)	1.00(0.99,1.01)	1.00(0.99,1.02)	1.00(0.99,1.02)	1.01(0.99,1.02)	1.01(0.99,1.02)	1.01(0.98,1.03)
**PM**_**2.5**_	36.00	1.00(0.99,1.01)	1.00(1.00,1.01)	**1.00(1.00,1.01)∗**	1.00(1.00,1.01)	1.00(1.00,1.00)	1.00(0.99,1.00)	0.99(0.98,1.00)
**PM**_**10**_	62.00	1.00(1.00,1.01)	**1.00(1.00,1.01)∗**	**1.00(1.00,1.01)∗**	**1.00(1.00,1.01)∗**	1.00(1.00,1.00)	1.00(1.00,1.00)	0.99(0.99,1.00)
**SO**_**2**_	8.00	1.02(0.98,1.05)	1.01(0.99,1.03)	1.00(0.98,1.02)	1.00(0.97,1.02)	1.00(0.98,1.01)	1.00(0.98,1.02)	1.00(0.97,1.04)
**NO**_**2**_	40.00	1.00(0.98,1.01)	**1.01(1.01,1.02)∗**	**1.03(1.02,1.04)∗**	**1.04(1.02,1.05)∗**	**1.03(1.02,1.04)∗**	**1.02(1.01,1.03)∗**	1.01(0.99,1.02)

Note: ∗ means p < 0.05; These results are presented the RR of DPAPV in multi -pollutant models when 10.0 μg/m3 increase for PM2.5, PM10, SO2, NO2, 0.1mg/m3 increase for CO, 10 °C increase for temperature, 10g/m3 increase for humidity, 10 mm increase for precipitation, 10 m/s increase for wind speed, 10 hPa increase for sea level pressure, 10m increase for visibility, and 10 increase for AQI.

### 3.4 WQS regression model

**Fig. 4:**
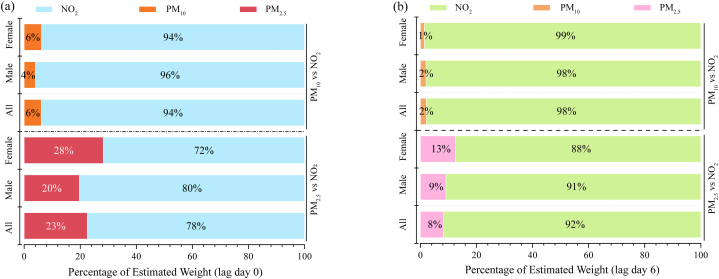
Estimated weights of air pollutants in the WQS regression analysis. (a) Comparing the estimated weights of NO_2_, PM_10_ and PM_2.5_ in three sub-groups in lag day 0. (b) Comparing the estimated weights of NO_2_, PM_2.5,_ and PM_10_ in three sub-groups in lag day 6.

PM_2.5_, PM_10_, and NO_2_ were important risk factors of the DPAPV. The WQS regression method was used to compare the relative importance of them. Because PM_2.5_, PM _10_, and NO_2_ were all positively associated with DPAPV (in Table S2, which showed correlations between environmental variables and DPAPV), their weights could be compared. Fig. 4 illustrates the estimated weights of PM_10_, PM_2.5,_ and NO_2_ in the WQS in different sub-groups on lag day 0 and lag day 6. In three sub-groups, NO_2_ showed a greater weight (over 70 %) than PM_2.5_ and PM_10_. Compared with females, the relative importance of NO_2_ was greater in males. Compared Fig. 4 (a)–(b), the weight of NO_2_ increased (4 %–14 %) with the lag time increase. Therefore, NO_2_ was the most important trigger of pediatric asthma among PM_2.5_, PM_10_, and NO_2_ which was consistent with the result of DLNM models. In addition, the relative importance of NO_2_ would increase with time passing (short-term lag time). The performances of NO_2_ were different between two genders, and males were susceptible to the harmful effects of NO_2_.

## 4 Discussion

In this study, the associations of environmental variables with DPAPV were evaluated by GAM-DLNM. NO_2_, PM_2.5_, and PM_10_ were important risk factors for pediatric asthma. Among them, NO_2_ had the greatest significant positive effect for a longer time (maximum RR appeared in lag day 3). Comparing the weight of NO_2_, PM_2.5_, and PM_10_ in the WQS model, the weights of NO_2_ were over 70 % in different sub-groups, and its weight increased as time passed. The weight of NO_2_ in males was also greater than that in females. Therefore, NO_2_ was the most important risk factor for DPAPV which obtained the greatest influence on lag day 3. Males were more adversely affected by NO_2_.

In this study, we found that the significant positive associations between air pollutants and pediatric asthma generally appeared in short lags and were weakened over longer lags (Table 2, Table 3), which was consistent with previous findings and could be explained by the cumulative and persistent effects of air pollutants and the delayed response of the immune system [15,16]. Among these air pollutants, the lag significantly affected the positive association between the levels of NO_2_ and the risk of pediatric asthma, while it did not affect the association between the levels of SO_2_ and this disease. Although its mechanism remains unclear, it can also be proven by a previous meta-analysis with a similar result [17].

NO_2_ is a ubiquitous air pollutant, which has a great threat to children's respiratory systems and this assertion is supported by a multitude of studies. Each year, there are about 2.56 million urban children with asthma worldwide are attributed to NO_2_ [18]. In 2019, a study estimated that there are about 637,000 new cases of pediatric asthma in urban areas in China were associated with long-term exposure to NO_2_, of which about 74 % were caused by outdoor NO_2_ [19]. In our results, NO_2_ had a strong positive correlation with DPAPV and the greatest impact of it appeared in the lag day 3. Both a study conducted in Hefei and a study conducted in Northern China identified similar correlations and short-term lag effects [20,21]. A nationwide birth cohort study from Denmark showed that early-life exposure to ambient high levels of NO_2_ could increase pediatric asthma incidence both in prenatal and postnatal periods [22]. An American study found that the NO_2_ which was primarily contributed by motor vehicles increased pediatric asthma emergency department visits [23]. NO2 increases the risk of pediatric asthma, incidence, daily visits, and emergency department visits, but the impact likely varies by different regions.

NO_2_ is more dangerous for pediatric asthma than PM because of its high permeability and high toxicity. Early experiments in the mouse can prove that NO_2_ could enhance and prolong inflammation of eosinophils and neutrophils, and prolonged airway hyperresponsiveness which worsens asthma control [24]. NO_2_ is almost insoluble in water and its molecule is very small which means it has high permeability in the lung. Therefore, it is easy to reach the distal airway through diffusion which will cause deep damage to the respiratory system (oxidative stress and long-term inflammation) [25,26]. As for PM, according to previous studies, NO_3_^−^, and SO_4_^2−^ were two main compounds of PM in Hangzhou [27]. NO_3_^−^ and SO_4_^2−^ would reduce the pH of the cell environment [28], which not only leads to decreased cell activity but also inflammation and mucus secretion in the airways. Mucus will prevent part of the PM from deeply penetrating the lung. Previous studies have also shown results consistent with ours [20,29,30].

The effect of NO_2_ on the risk of pediatric asthma also had sex differences, and NO_2_ was a relatively more important air pollutant in boys than girls. It may be caused by the physiological differences. Compared to girls, lung function development was more erratic in boys, with slower rates of development and maturity [31]. The incompletely developed lung protective mechanisms, such as bronchial mucus and cilia cleansing, make them vulnerable to early NO₂ exposure. In addition, boys are more likely to suffer from pulmonary dysanapsis (mismatches between the lungs and airways), which makes them more sensitive to airborne irritants [32].

Although PM_2.5_ and PM_10_ did not have the same importance as NO_2_ in this study, they also had a significant positive association with the risk of pediatric asthma. Its primary mode of toxicity is inhalation, which allows it to enter the circulation and lungs and cause irritation or injury [33,34]. Therefore, the adverse effect of PM on pediatric asthma is also being noted. To date, there were numerous studies aimed at the effect of PM on this disease, and they also found exposure to the PM, especially compositions of BC, OM, and SO_4_^2−^, was significantly positively associated with the increased risk of pediatric asthma [35,36]. It is worth noting that compared with NO_2_, more studies explored the long-term effect of PM because of the high correlation between NO_2_ and other air pollutants [37]. A long-term observational study showed that long-term exposure to PM_10-2.5_ was associated with increased asthma diagnosis prevalence in children [38]. Another study in Kenya also found that high levels of NO_2_ increase the risk for acute respiratory problems (such as asthma), but they found no short-term lag effect which was inconsistent with our result [39].

The main strengths of this study are as follows: First, this is the first study to use both GAM-DLNM and WQS models to explore the most important risk factor for pediatric asthma in common environmental variables, which has relevant implications for parents and doctors to control pediatric asthma in Hangzhou. Second, we also considered the interaction and causality between environmental variables in multi-variable models to make the results more convincing. Third, large sample in this study. There are 313,296 samples between 2014 and 2021 in this study.

Inevitably, there are some limitations to the results of this study. Firstly, this was a single-center study. The willingness of outpatient visits to this hospital may have affected the results. This hospital was the center of children's health care in Zhejiang Province, and its outpatient visits are representative of the children in Hangzhou. However, people are more inclined to have treatment at the nearest hospital (even the private clinics or community-based health care) during an asthma emergency, which may lead to an underestimated RR in this study. Especially during 2019 and 2021, these places may become the first choice for patients due to the impact of the COVID-19 (coronavirus disease 2019) pandemic. Secondly, the application of the WQS regression model assumed that every patient has the same exposure, which might underestimate individual variability in health outcomes. Thirdly, we have controlled some confounding factors (such as weekends, visit month, and visit year), but the other factors, such as family income, genetics, and family address, were not controlled in this study, which might lead to GAM-DLNM misestimating the risk of pediatric asthma. However, questionnaires and genetic tests are expensive and time-consuming, and patients will refuse to complete them because of privacy concerns.

## 5 Conclusions

NO_2_ is the most important risk factor for pediatric asthma (its adverse effect reached maximum at lag day 3), and it has a greater effect on males. These results are important for parents to realize the influence of air pollution on pediatric asthma, and help them to prevent the recurrence of asthma in their children to some extent. For the government, it can be used as evidence to continue energy conservation and emission reduction to improve air quality.

## Funding source

This work was supported by the 10.13039/501100001809National Natural Science Foundation of China [81871456, 82000030].

## CRediT authorship contribution statement

**Yuqing Feng:** Writing – original draft, Visualization, Validation, Investigation, Data curation. **Xin Yang:** Validation, Investigation, Data curation. **Yingshuo Wang:** Validation, Methodology, Conceptualization. **Lei Wu:** Validation, Methodology, Conceptualization. **Qiang Shu:** Visualization, Validation, Supervision, Project administration, Methodology, Conceptualization. **Haomin Li:** Visualization, Validation, Supervision, Project administration, Methodology, Funding acquisition, Conceptualization.

## Declaration of competing interest

The authors declare that they have no known competing financial interests or personal relationships that could have appeared to influence the work reported in this paper.

## Footnotes

Appendix A Supplementary data to this article can be found online at https://doi.org/10.1016/j.heliyon.2024.e37837.

## References

[1] Bateson T.F., Schwartz J. (2008). Children's response to air pollutants. J. Toxicol. Environ. Health.

[2] C.C. for D.C. and P. Institute of Environmental Health and Related Product Safety, N.C.G. on C. Asthma, C.C. for D.C. and Prevention (2013).

[3] Mims J.W. (2015). Asthma: definitions and pathophysiology. Int Forum Allergy Rhinol.

[4] Xiang L., Zhao J., Zheng Y., Liu H., Hong J., Bao Y., Chen A., Deng L., Ji W., Zhong N., Shen K. (2016). Uncontrolled asthma and its risk factors in Chinese children: a cross-sectional observational study. J. Asthma.

[5] Zhou D., Lin Z., Liu L., Qi J. (2021). Spatial-temporal characteristics of urban air pollution in 337 Chinese cities and their influencing factors. Environ. Sci. Pollut. Control Ser..

[6] Zhou A., Li J. (2021). Air pollution and income distribution: evidence from Chinese provincial panel data. Environ. Sci. Pollut. Control Ser..

[7] Liu L., Liu C., Chen R., Zhou Y., Meng X., Hong J., Cao L., Lu Y., Dong X., Xia M., Ding B., Qian L., Wang L., Zhou W., Gui Y., Zhang X. (2021). Associations of short-term exposure to air pollution and emergency department visits for pediatric asthma in Shanghai, China. Chemosphere.

[8] Zhang Y., Wu Z., Gou K., Wang R., Wang J. (2021). The impact of air pollution on outpatient visits of children with asthma in xi’an, China. Wilderness Environ. Med..

[9] Zhang J., Dai J., Yan L., Fu W., Yi J., Chen Y., Liu C., Xu D., Wang Q. (2016). Air pollutants, climate, and the prevalence of pediatric asthma in urban areas of China. Biomed Res Int 2016.

[10] Gasparrini A., Scheipl F., Armstrong B., Kenward M.G. (2017). A penalized framework for distributed lag non-linear models. Biometrics.

[11] Carrico C., Gennings C., Wheeler D.C., Factor-Litvak P. (2015). Characterization of weighted quantile sum regression for highly correlated data in a risk analysis setting. J. Agric. Biol. Environ. Stat..

[12] Eggers S., Bixby M., Renzetti S., Curtin P., Gennings C. (2023). Human microbiome mixture analysis using weighted quantile sum regression. Int J Environ Res Public Health.

[13] Stewart G.D., Phipps S., Little B., Leveckis J., Stolzenburg J.U., Tolley D.A., McNeill S.A., Riddick A.C.P. (2012). Description and validation of a modular training system for laparoscopic nephrectomy. J. Endourol..

[14] Dong B.R., Gu X.Q., Chen H.Y., Gu J., Pan Z.G. (2021). Development and validation of a nomogram to predict frailty progression in nonfrail Chinese community-living older adults. J. Am. Med. Dir. Assoc..

[15] Ma R., Zhang G., Kong Y., Jia S. (2023). Regional heterogeneity in short-term associations of meteorological factors, air pollution, and asthma hospitalizations in Guangxi, China. Publ. Health.

[16] Hsu S.C., Chang J.H., Lee C.L., Huang W.C., Hsu Y.P., Te Liu C., Jean S.S., Huang S.K., Hsu C.W. (2020). Differential time-lag effects of ambient PM2.5 and PM2.5-bound PAHs on asthma emergency department visits. Environ. Sci. Pollut. Control Ser..

[17] Orellano P., Quaranta N., Reynoso J., Balbi B., Vasquez J. (2017). Effect of outdoor air pollution on asthma exacerbations in children and adults: systematic review and multilevel meta-analysis. PLoS One.

[18] Achakulwisut P., Brauer M., Hystad P., Anenberg S.C. (2019). Global, national, and urban burdens of paediatric asthma incidence attributable to ambient NO(2) pollution: estimates from global datasets. Lancet Planet. Health.

[19] Hu Y., Ji J.S., Zhao B. (2022). Restrictions on indoor and outdoor NO(2) emissions to reduce disease burden for pediatric asthma in China: a modeling study. Lancet Reg Health West Pac.

[20] Zhang Y., Ni H., Bai L., Cheng Q., Zhang H., Wang S., Xie M., Zhao D., Su H. (2019). The short-term association between air pollution and childhood asthma hospital admissions in urban areas of Hefei City in China: a time-series study. Environ. Res..

[21] Zhao Y., Kong D., Fu J., Zhang Y., Chen Y., Liu Y., Chang Z., Liu Y., Liu X., Xu K., Jiang C., Fan Z. (2021). Increased risk of hospital admission for asthma in children from short-term exposure to air pollution: case-crossover evidence from northern China. Front. Public Health.

[22] Pedersen M., Liu S., Zhang J., Andersen Z.J., Brandt J., Budtz-Jørgensen E., Bønnelykke K., Frohn L.M., Andersen A.M.N., Ketzel M., Khan J., Stayner L., Brunekreef B., Loft S. (2023). Early-life exposure to ambient air pollution from multiple sources and asthma incidence in children: a nationwide birth cohort study from Denmark. Environ. Health Perspect..

[23] Khatri S.B., Newman C., Hammel J.P., Dey T., Van Laere J.J., Ross K.A., Rose J.A., Anderson T., Mukerjee S., Smith L., Landis M.S., Holstein A., Norris G.A. (2021).

[24] Poynter M.E., Persinger R.L., Irvin C.G., Butnor K.J., van Hirtum H., Blay W., Heintz N.H., Robbins J., Hemenway D., Taatjes D.J., Janssen-Heininger Y. (2006). Nitrogen dioxide enhances allergic airway inflammation and hyperresponsiveness in the mouse. Am. J. Physiol. Lung Cell Mol. Physiol..

[25] Greenberg N., Carel R.S., Derazne E., Bibi H., Shpriz M., Tzur D., Portnov B.A. (2016). Different effects of long-term exposures to SO2 and NO2 air pollutants on asthma severity in young adults. J. Toxicol. Environ. Health.

[26] Strassmann A., de Hoogh K., Roosli M., Haile S.R., Turk A., Bopp M., Puhan M.A., Swiss National Cohort Study G. (2021). NO2 and PM2.5 exposures and lung function in Swiss adults: estimated effects of short-term exposures and long-term exposures with and without adjustment for short-term deviations. Environ. Health Perspect..

[27] Zhang H.H., Li Z., Liu Y., Xinag P., Cui X.Y., Ye H., Hu B.L., Lou L.P. (2018). Physical and chemical characteristics of PM(2.5) and its toxicity to human bronchial cells BEAS-2B in the winter and summer. J. Zhejiang Univ. - Sci. B.

[28] Huang M., Kang Y., Wang W., Chan C.Y., Wang X., Wong M.H. (2015). Potential cytotoxicity of water-soluble fraction of dust and particulate matters and relation to metal(loid)s based on three human cell lines. Chemosphere.

[29] Lu P., Zhang Y., Lin J., Xia G., Zhang W., Knibbs L.D., Morgan G.G., Jalaludin B., Marks G., Abramson M., Li S., Guo Y. (2020). Multi-city study on air pollution and hospital outpatient visits for asthma in China. Environ Pollut.

[30] Zhang S., Li G., Tian L., Guo Q., Pan X. (2016). Short-term exposure to air pollution and morbidity of COPD and asthma in East Asian area: a systematic review and meta-analysis. Environ. Res..

[31] Chowdhury N.U., Guntur V.P., Newcomb D.C., Wechsler M.E. (2021). Sex and gender in asthma. Eur. Respir. Rev..

[32] Pignataro F.S., Bonini M., Forgione A., Melandri S., Usmani O.S. (2017). Asthma and gender: the female lung. Pharmacol. Res..

[33] Jia H., Liu Y., Guo D., He W., Zhao L., Xia S. (2021). PM2.5-induced pulmonary inflammation via activating of the NLRP3/caspase-1 signaling pathway. Environ. Toxicol..

[34] Park E.J., Yoon C., Han J.S., Lee G.H., Kim D.W., Park E.J., Lim H.J., Kang M.S., Han H.Y., Seol H.J., Kim K.P. (2021). Effect of PM10 on pulmonary immune response and fetus development. Toxicol. Lett..

[35] Zhang Y., Yin Z., Zhou P., Zhang L., Zhao Z., Norbäck D., Zhang X., Lu C., Yu W., Wang T., Zheng X., Zhang L., Zhang Y. (2022). Early-life exposure to PM2.5 constituents and childhood asthma and wheezing: findings from China, Children, Homes, Health study. Environ. Int..

[36] Wu J., Zhong T., Zhu Y., Ge D., Lin X., Li Q. (2019). Effects of particulate matter (PM) on childhood asthma exacerbation and control in Xiamen, China. BMC Pediatr..

[37] Costa S., Ferreira J., Silveira C., Costa C., Lopes D., Relvas H., Borrego C., Roebeling P., Miranda A.I., Paulo Teixeira J. (2014). Integrating health on air quality assessment - review report on health risks of two major european outdoor air pollutants: PM and NO2. J. Toxicol. Environ. Health B Crit. Rev..

[38] Keet C.A., Keller J.P., Peng R.D. (2018). Long-term coarse particulate matter exposure is associated with asthma among children in medicaid. Am. J. Respir. Crit. Care Med..

[39] Larson P.S., Espira L., Glenn B.E., Larson M.C., Crowe C.S., Jang S., O’neill M.S. (2022). Long-term PM2.5 exposure is associated with symptoms of acute respiratory infections among children under five years of age in Kenya, 2014. Int J Environ Res Public Health.

