# Supplementary material for: The short-term association between environmental variables and daily pediatric asthma patient visits in Hangzhou, China: A time-series study
Source: Heliyon. 2024 Sep 13;10(18):e37837. doi: 10.1016/j.heliyon.2024.e37837 (PMC11425122; doi:10.1016/j.heliyon.2024.e37837)
Supplement: Multimedia component 1 [file mmc1.docx]

**Table S1** Descriptive statistics of meteorological variables and air pollutants

|  | **Min** | **P25** | **Median** | **P75** | **Max** | **Mean** | **SD** |
| --- | --- | --- | --- | --- | --- | --- | --- |
| **Temperature (****°C)** | -4.20 | 10.80 | 19.10 | 25.50 | 35.30 | 18.31 | 8.60 |
| **Humidity (g/m^3^)** | 27.16 | 64.26 | 74.02 | 83.37 | 99.63 | 73.19 | 13.51 |
| **Precipitation (mm)** | 0.00 | 0.00 | 0.00 | 3.60 | 134.00 | 4.72 | 11.26 |
| **Wind speed (m/s)** | 7.20 | 14.40 | 18.00 | 21.60 | 54.00 | 17.50 | 5.52 |
| **Sea level pressure (hPa)** | 983.60 | 1008.40 | 1016.50 | 1023.60 | 1042.40 | 1016.42 | 9.24 |
| **Visibility (m)** | 1.30 | 5.40 | 7.70 | 10.00 | 14.50 | 7.63 | 2.78 |
| **AQI** | 18.00 | 55.00 | 74.00 | 97.00 | 283.00 | 80.06 | 33.97 |
| **PM_2.5_ (****μg/m^3^)** | 0.00 | 24.00 | 36.00 | 55.00 | 233.00 | 42.98 | 27.32 |
| **PM_10_ (μg/m^3^)** | 0.00 | 42.00 | 62.00 | 91.00 | 305.00 | 71.32 | 39.85 |
| **SO_2_ (μg/m^3^)** | 3.00 | 6.00 | 8.00 | 13.00 | 78.00 | 10.71 | 6.98 |
| **NO_2_ (μg/m^3^)** | 6.00 | 30.00 | 40.00 | 53.00 | 119.00 | 42.05 | 16.54 |
| **CO (mg/m^3^)** | 0.30 | 0.60 | 0.80 | 0.90 | 2.00 | 0.82 | 0.23 |

**Table S2** Spearman correlations between Environmental Variables and DPAPV

| **Variable****s** | **Correlation** | **P value** | **Variables** | **Correlation** | **P value** |
| --- | --- | --- | --- | --- | --- |
| **Temperature** | 0.017 | 0.350 | **AQI** | 0.145 | <0.001 |
| **Humidity** | -0.022 | 0.238 | **PM_2.5_** | 0.195 | <0.001 |
| **Precipitation** | -0.046 | 0.014 | **PM_10_** | 0.185 | <0.001 |
| **Wind Speed** | -0.062 | <0.001 | **SO_2_** | 0.338 | <0.001 |
| **Sea level pressure** | 0.067 | <0.001 | **NO_2_** | 0.258 | <0.001 |
| **Visibility** | -0.010 | <0.001 | **CO** | 0.245 | <0.001 |

**
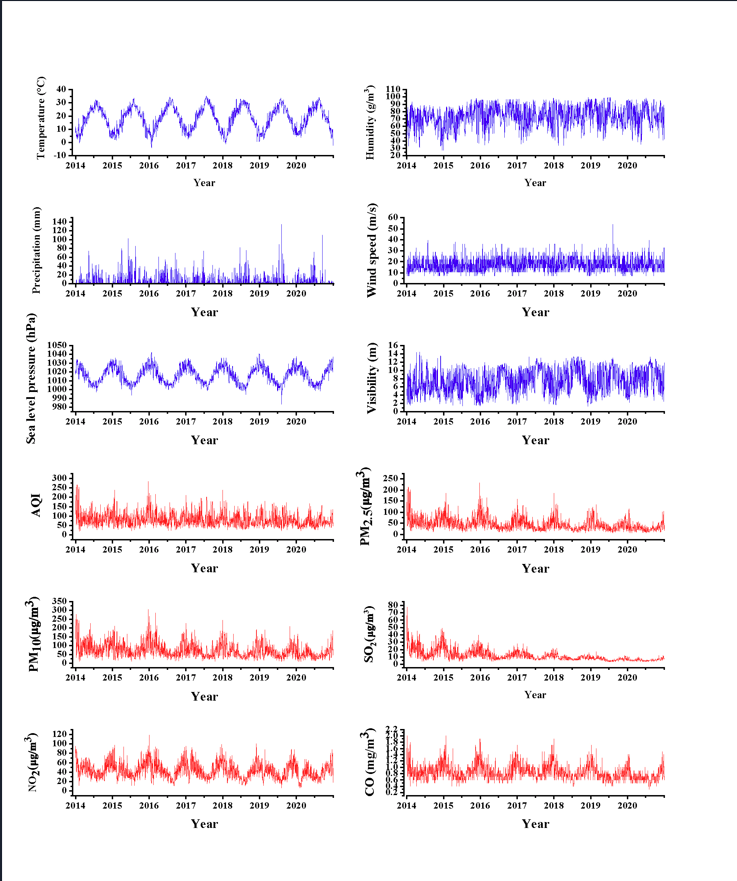
**

**Fig S1** Daily meteorological variable, and air pollutants concentrations between January 1, 2014, and December 31, 2021
